# DAMP production by human islets under low oxygen and nutrients in the presence or absence of an immunoisolating-capsule and necrostatin-1

**DOI:** 10.1038/srep14623

**Published:** 2015-09-30

**Authors:** Genaro A. Paredes-Juarez, Neha M. Sahasrabudhe, Reina S. Tjoelker, Bart J. de Haan, Marten A. Engelse, Eelco J. P. de Koning, Marijke M. Faas, Paul de Vos

**Affiliations:** 1 University of Groningen, University Medical Center Groningen, Department of Pathology and Medical Biology, Section of Immunoendocrinology, Groningen, 9713 GZ, The Netherlands; 2 Department of Nephrology, Leiden University Medical Center, Leiden, 2300 RC, The Netherlands

## Abstract

In between the period of transplantation and revascularization, pancreatic islets are exposed to low-oxygen and low-nutrient conditions. In the present study we mimicked those conditions *in vitro* to study the involvement of different cell death processes, release of danger-associated molecular patterns (DAMP), and associated *in vitro* immune activation. Under low-oxygen and low-nutrient conditions, apoptosis, autophagy and necroptosis occur in human islets. Necroptosis is responsible for DAMP-release such as dsDNA, uric acid, and HMGB1. The sensors of the innate immune system able to recognize these DAMPs are mainly TLR, NOD receptors, and C-type lectins. By using cell-lines with a non-functional adaptor molecule MyD88, we were able to show that the islet-derived DAMPs signal mainly via TLR. Immunoisolation in immunoprotective membranes reduced DAMP release and immune activation via retention of the relative large DAMPs in the capsules. Another effective strategy was suppressing necroptosis using the inhibitor nec-1. Although the effect on cell-survival was minor, nec-1 was able to reduce the release of HMGB1 and its associated immune activation. Our data demonstrate that in the immediate post-transplant period islets release DAMPs that *in vitro* enhance responses of innate immune cells. DAMP release can be reduced *in vitro* by immunoisolation or intervention with nec-1.

Islet transplantation has the potential to supply patients with type 1 diabetes with an endogenous insulin source that regulates the glucose levels on a minute-to-minute level. A major breakthrough was reported in 2000 when insulin independence in a series of seven patients for a period of a year was reported^1^. Despite this breakthrough, the approach has not yet reached the stage of large-scale clinical application. The reasons for this is the shortage of organ donors and the relative short duration of graft survival with inevitable lost in functionality. Two years after transplantation, less than 50% of the patients is still insulin-independent^2^ and five years following transplantation this percentage is not more than 10%^3^. Although during recent years some groups have been more successful^4^, the overall conclusion has to be that many factors remain to be improved in the islet-transplantation procedures before large-scale clinical application is a realistic option.

During recent years many groups have focused on the identification of factors determining success or failure of islet grafts. Factors that have been implicated in the low success rates are the instant blood mediated inflammatory reaction^5^,^6^,^7^, alloreactivity^5^, recurrent autoimmunity^5^, NK-cell cytotoxicity^8^, inadequacy of the liver as transplantation site^9^, local and systemic T cell responses and macrophage recruitment^10^, and insufficient revascularization^11^. In most cases, however, the focus has been on external, host factors that influence islet-survival and not on mechanisms by which the islets themselves contribute to their own destruction. Islets are potent producers of all types of bioactive molecules and may enhance inflammatory responses against themselves^11^.

Danger-associated molecular patterns (DAMPs) are molecules from the intracellular space that upon release to the extracellular space are recognized as alarm signals^12^. DAMPs can induce severe inflammation and are part of the pathogenesis of aseptic or sterile inflammation^13^. DAMPs are recognized by pattern recognition receptors (PRRs), mainly by Toll-like receptors (TLRs), and induce an inflammation cascade even when present in low amounts^14^,^15^. Examples of DAMPs are high mobility group box 1 protein (HMGB1) that in normal conditions play a role in the winding of DNA and promotes assembly of proteins^12^,^16^. Another example is heat shock protein 70 (HSP70) that has been implicated in failure of transplanted kidneys^17^. HSP70 effectively promotes recruitment of immune cells^17^,^18^,^19^. Other molecules that are studied and have been identified as DAMPs are uric acid and DNA-fragments^14^.

DAMPs are released during ischemia-reperfusion damage^20^ as the consequence of cells that die by necrosis and necroptosis^15^,^18^,^21^,^22^. As islets undergo a relative long phase of ischemia in between the period of implantation and revascularization it is plausible that also DAMPs play a role in islet-graft failure^23^. It has also been demonstrated that islets are able to release the monocyte chemoattractant protein-1 (MCP-1) which influencing the survival of grafts^24^. Indeed, in transplantation-research, it becomes currently recognized that DAMPs might play a crucial role in the responses against grafts^14^. However, to our best knowledge, DAMPs produced by islets have not been studied in detail.

Therefore, in the present study human pancreatic islets were subjected to suboptimal culture conditions mimicking the circumstances islets meet directly after transplantation. To this end, islets were cultured under hypoxic conditions or low-nutrient conditions to mimic the phase in which islets are not vascularized. Normal culture conditions served as control. The release of DAMPs was studied at day 1 and 7 after the start culture, which are critical days for graft loss after transplantation *in vivo*^11^. The DAMPs, HMGB1, DNA and uric acid were measured in the culture supernatant. Moreover, culture supernatants were co-incubated with a THP1 reporter cell-line expressing TLRs and a THP1 cell-line with a truncated and inactive MyD88 signaling pathway. These experiments were also done with islets immunoisolated in alginate-PLL-alginate (APA) capsule and a necroptosis inhibitor to investigate the efficacy of encapsulation or pharmaceutical intervention in preventing DAMP-induced immune activation by human pancreatic islets.

## Results

### Islet-derived molecules contribute to TLR-dependent immune activation

In order to investigate whether supernatant of islets cultured under the combination of low nutrients and or hypoxia induce immune activation, the supernatant of pancreatic islets were co-incubated with THP1-XBlue**™**-MD2-CD14 cell-line. This cell-line contains all TLRs as well as other PRRs^25^,^26^. To study whether the signaling is dependent on TLRs, and not on other PRRs, we subsequently tested the supernatants on a THP1 cell expressing a non-functional form of the TLR adapter MyD88 (Supplemental Fig. S1).

**Figure 1:**
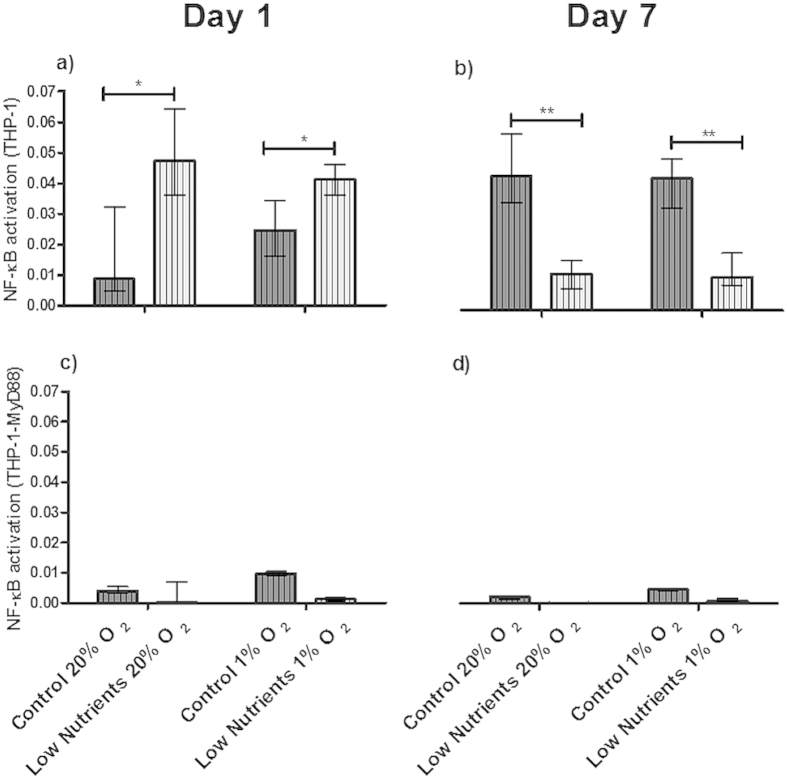
TLR dependency of the activation of THP1 cells by islet derived molecules. THP1 cells were stimulated with supernatant of human islets incubated for 1 (**a**) and 7 (**b**) days under control and low-nutrition conditions at 20% and 1% of oxygen. NF-κB/AP-1 activation was enhanced under low-nutrients and low oxygen conditions in a TLR fashion, since this activation was virtually absent in THP1 MyD88-deficient cell lines at 1 (**c**) or 7 (**d**) days of incubation. Values are presented as median ± IQR (n = 4 separate batches of human islets). A *p *< 0.05 was consider statistical significant (*p < 0.05; **p < 0.01). LPS (10 μg/ml) and Tri-DAP (10 μg/ml) were used as positive control for THP1 and THP1 MyD88-deficient cell lines respectively.

As shown in Fig. 1, even under control conditions immune-activation of THP1-XBlue**™**-MD2-CD14 was observed at 1 day (Fig. 1a). This activation was more enhanced by low-nutrient conditions than by hypoxia. The effect of low-nutrient conditions was reduced after 7 days of culturing (Fig. 1b) (*p* < 0.05). The reduction, can be explained by survival of a lower number of islet-cells that contribute to DAMPs production as will be presented in the next section, in which almost 40% of the cells are lost due to the harsh culture circumstances. The activation was TLR-dependent as the activation was profoundly reduced when the same supernatant was co-incubated with THP1 cells expressing the non-functional MyD88 under combined hypoxic and low-nutrient conditions (Fig. 1c,d).

### Viability and cell-death processes involved

**Figure 2:**
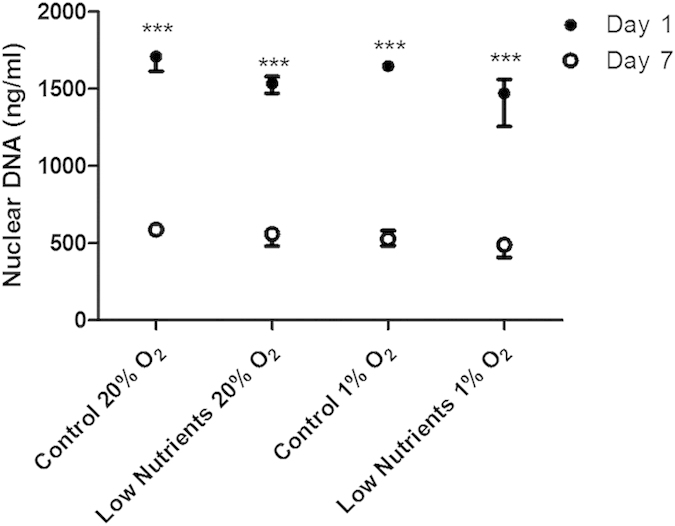
Nuclear DNA content of human islet cultures. DNA was quantified after isolation of nuclei of cultured islets. Islets were incubated for 1 and 7 days under control conditions or under low nutrient condition in combination with 20% or 1% of oxygen. Values are presented as median ± IQR (n = 4 separate batches of human islets). A *p *< 0.05 was considered statistical significant (***p < 0.001).

To determine the loss of islet-cells as the consequence of the different culture conditions the nuclear DNA on day 1 and 7 was studied as a measure for surviving cells. Under normal control conditions and low-nutrient exposure in normoxic conditions an approximate reduction of 35.45 ± 1.97% and 35.43 ± 3.91% of the cells was observed. This reduction was 31.43 ± 5.43% under hypoxia but normal nutrient conditions and when islets were exposed to the combination hypoxia and low-nutrient conditions a reduction of 32.15 ± 5.27% was observed (Fig. 2). All the reductions in nuclear DNA were statistically significant (*p *< 0.001).

**Figure 3:**
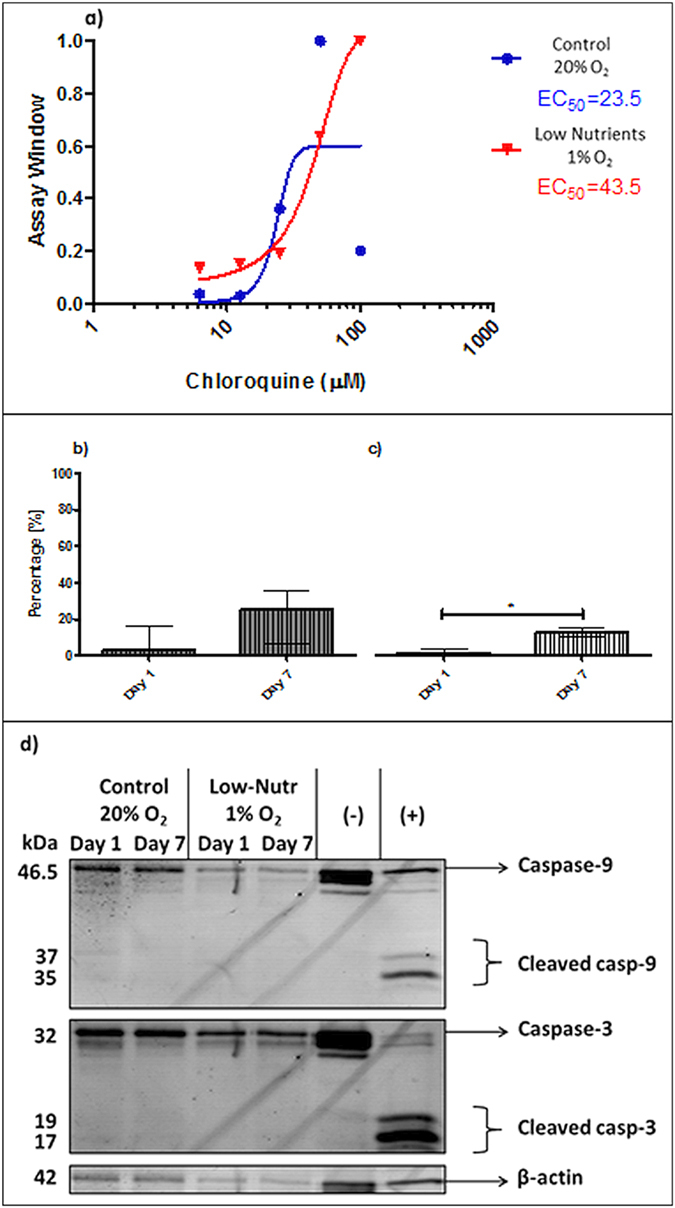
Autophagy and apoptosis in human pancreatic islets. TR-FRET acceptor in human islets incubated under control conditions in normoxia or under low nutrient conditions and hypoxia (**a**). Assay windows values are plotted as mean (n = 2 duplicate wells for each concentration of CQ). Percentage of apoptotic islets were calculated as positive islets stained with Annexin V cultured in control normoxic conditions and (**b**) or hypoxic low nutrients (**c**). Representative western blot picture for caspase-3 and −9 (**d**). Values are presented as median ± IQR. A *p *< 0.05 was considered significant (*p < 0.05).

Islets were subsequently subjected to studies for autophagy and apoptosis (Fig. 3). The autophagy TR-FRET assay serves to quantify autophagosomes in islets^27^. As shown in Fig. 3a, islets cultured under low-nutrient and hypoxic conditions have almost twice as much autophagic bodies (EC_50 _= 43.5) when compared to control conditions (EC_50 _= 23.5). Primary apoptosis was quantified by counting the number of Annexin V positive cells and was found to be statistically significant enhanced by low-nutrient hypoxic conditions (*p *< 0.05) (Fig. 3c) but not by control normoxic conditions (Fig. 3b). As Annexin V staining identifies apoptotic cells in an early stage, the islets were also subjected to caspase-3 and −9 analysis by western blot, to demonstrate the more irreversible stage of apoptosis^28^. After incubation under low-nutrients hypoxic and control normoxic conditions, full caspase-3 and −9 was found but not their cleaved form (Fig. 3d). This indicates an active process of cellular death towards necrosis rather than secondary apoptosis^29^,^30^.

**Figure 4:**
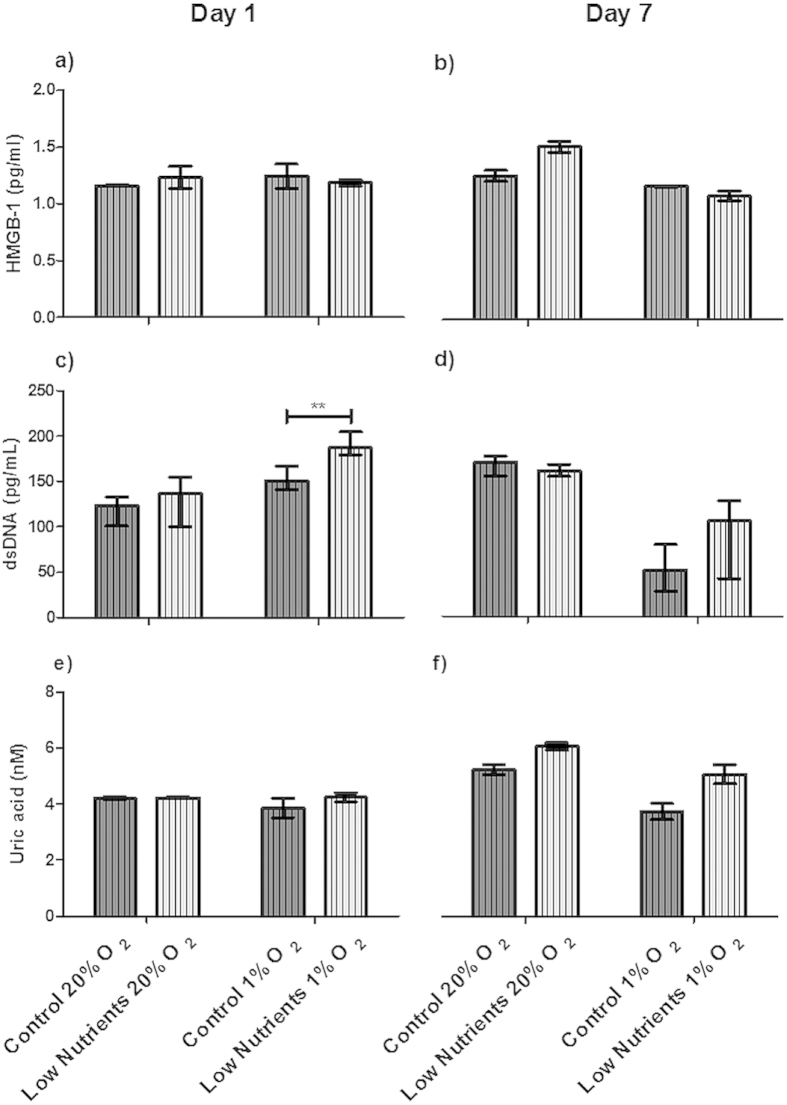
Danger-associated molecular patterns (DAMPs) released by free human islets. Human islets were incubated for 1 (**a**,**c**,**e**) or 7 (**b**,**d**,**f**) days under control and low nutrient conditions at 20% and 1% of oxygen. DAMPs produced by islets were high mobility group protein B1 (HMGB1; (**a**,**b**)), double stranded DNA (dsDNA; (**c**,**d**)), and uric acid (**e**,**f**). Values are presented as median ± IQR (n = 4 separate batches of human islets). A *p *< 0.05 was considered statistically significant (**p < 0.01).

Release of DAMPs is a mean for quantification of the involvement of necrosis and necroptosis^18^,^22^,^23^. The most commonly studied DAMPs in necrosis or necroptosis are heat shock protein 70 (HSP70), double stranded DNA (dsDNA), uric acid, and high mobility group box 1 (HMGB1)^19^,^21^,^22^. In none of the conditions tested we observed HSP70 release. This was different with HMGB1, uric acid, and dsDNA. These DAMPs were constitutively present in culture media even under control conditions. HMGB1 release was found under all culture conditions without any significant difference between different culture conditions (Fig. 4a,b). This release is possibly due to loss in cell-viability.

The dsDNA concentration increased significantly (*p *< 0.01) after 1 day of hypoxic culture under low nutrients when compared with control conditions (Fig. 4c). Also, after culture for 7 days under hypoxic conditions (Fig. 4d), low values of dsDNA can be observed, this can be explained again by a lower number of cells surviving the severe conditions.

Uric acid was present under all conditions but was not statistically significant enhanced by low nutrient or hypoxic conditions (Fig. 4e,f).

### Immunoisolation of pancreatic islets reduce immune activation

**Figure 5:**
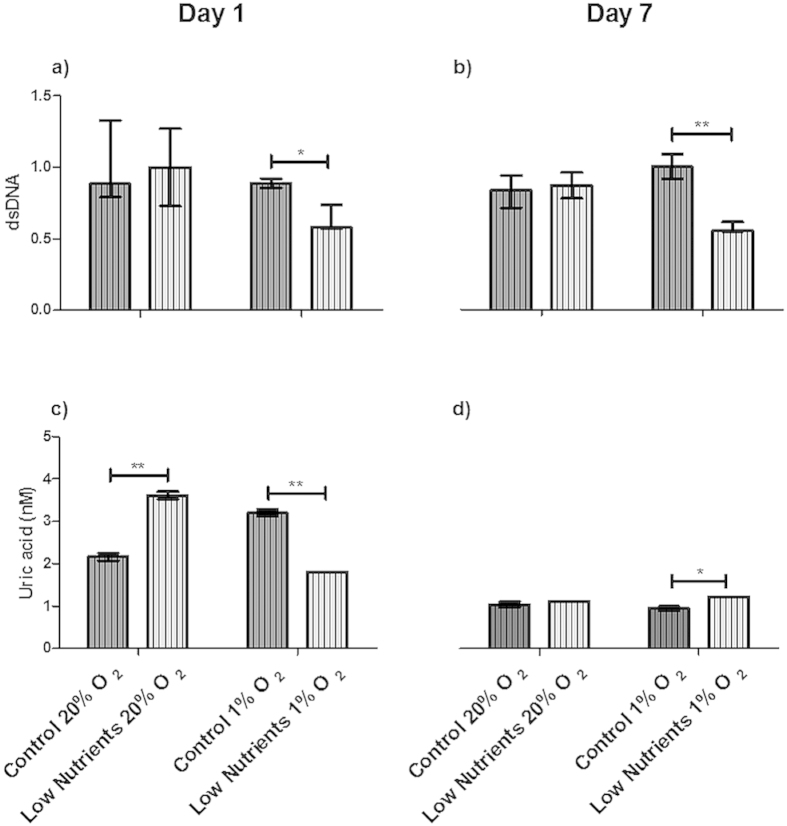
Reduction of danger-associated molecular patterns (DAMPs) from immunoisolated islets in alginate-based microcapsules. Supernatant of encapsulated pancreatic human islets incubated for 1 (**a**,**c**) and 7 (**b**,**d**) days under control and low nutrients conditions at 20% and 1% of oxygen. DAMPs found were double stranded DNA (dsDNA; (**a**,**b**)), and uric acid (**c**,**d**). High mobility group protein B1 (HMGB1) was not found in encapsulated islets and therefore not shown in the graph. Values are presented as median ± IQR (n = 4 separate batches of human islets). a *p *< 0.05 was consider statistical significant (*p < 0.05; **p < 0.01).

Immunoisolation as a mean to prevent rejection of islets is gaining much attention by the scientific community^7^,^9^. DAMPs are relatively large structures that should be retained by the membranes. To confirm this, human islets were encapsulated in alginate-PLL-alginate (APA) capsules and cultured under the same low-nutrient and hypoxic conditions. Supernatant was subsequently incubated with THP1-XBlue**™**-MD2-CD14. The induced responses were up to tenfold lower when compared to free islets after 1 day of incubation under all the different conditions (supplemental Fig. S2) indicating that encapsulation prevents the release of some DAMPs. To confirm that, we quantified the release of DAMPs by encapsulated islets after incubation. Interestingly, HMGB1 was absent under all incubation conditions. We detected dsDNA under low (Fig. 5a) or normal nutrient conditions (Fig. 5b), and under hypoxia or normoxia, with no statistical significant differences. Uric acid was statistically significant increased (*p *< 0.05) on day 1 and 7 under all the conditions with low nutrient availability (Fig. 5c,d).

### Necrostatin -1 promotes cell survival and reduces DAMPs release

**Figure 6:**
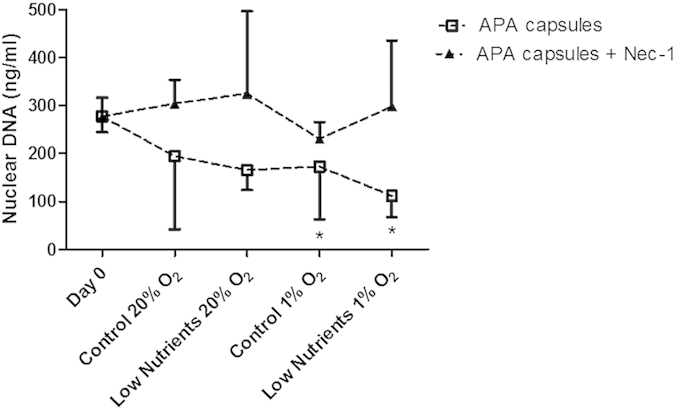
Comparison of nuclear DNA content between encapsulated islets with or without nec-1. Nuclei of control encapsulated and encapsulated nec-1 human islets were isolated for quantification of DNA before and after incubation for 7 days under control and low nutrients at 20% and 1% of oxygen. Human islets encapsulated in conventional APA system contained statistical significantly lower amounts of DNA after incubation under control and low nutrients at 1% of oxygen (*p < 0.05) when compared with capsules at day 0. Values are presented as median ± IQR (n = 4 separate batches of human islets).

Since necroptosis is one of the main cell-death processes responsible for release of DAMPs, we studied the effect of nec-1, a potent inhibitor of necroptosis^22^, on cell survival after 7 days of culture in the different conditions. At day 0, islets contained 279.54 ± 37.39 ng/ml of nuclear DNA. The DNA content decreased under the different culture conditions, which could partly be prevented by addition of nec-1 to the capsules. This however only reached statistical significance when cultured in 1% of oxygen in the presence or absence of low-nutrient conditions when compared with day 0 (Fig. 6).

**Figure 7:**
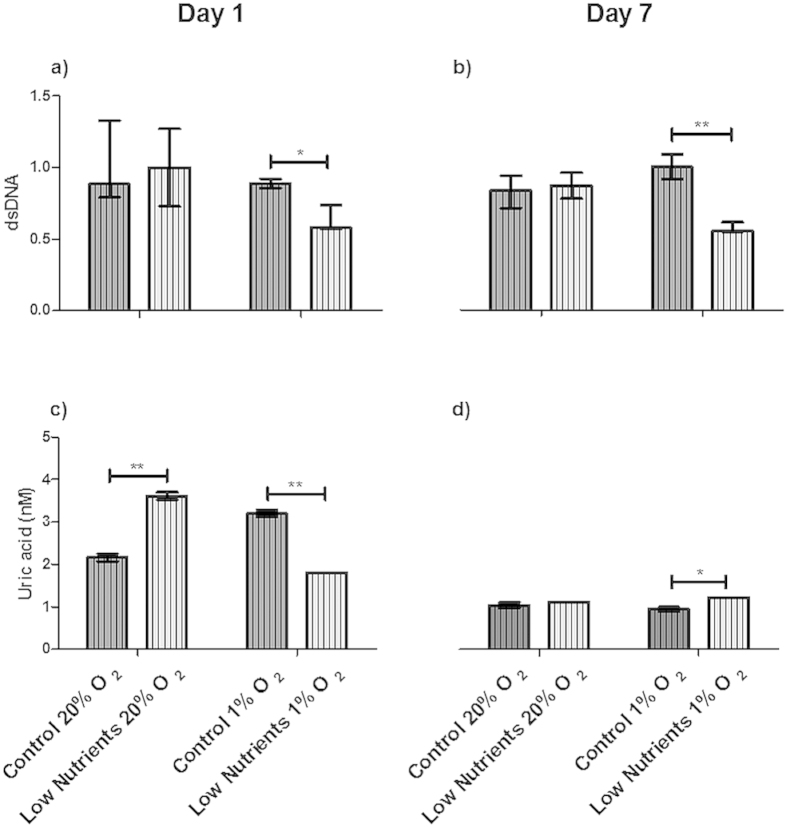
Reduction of danger-associated molecular patterns (DAMPs) by nec-1 in encapsulated human islets. Supernatant of encapsulated human islets with nec-1 incubated for 1 (**a**,**c**) and 7 (**b**,**d**) days under control and low nutrients at 20% and 1% of oxygen. DAMPs found were double stranded DNA (dsDNA; (**a**,**b**)), and uric acid (**c**,**d**). DsDNA was normalized. High mobility group protein B1 (HMGB1) was not found in supernatant of encapsulated islets and is therefore not shown in the graph. Values are presented as median ± IQR (n = 4 separate batches of human islets). a *p *< 0.05 was consider significant (*p < 0.05; **p < 0.01).

To determine whether nec-1 addition also reduces immune activation and DAMPs release, the effects where studied on THP1 cells and on DAMP production. Supernatant of encapsulated islets treated with nec-1 provoked a lower NF-κB activation in THP1 cells than the non-treated encapsulated islets. This was observed under low nutrition conditions combined with normoxia and in low nutrition conditions under hypoxia when compared with free islets after 1 day of incubation (supplemental Fig. S3). This reduction in immune-activation was associated with lower release (*p *< 0.05) of the DAMP dsDNA also under low-nutrients and hypoxic conditions after 1 and 7 days of incubation (Fig. 7a,b). Uric acid was also reduced (*p *< 0.001) by nec-1 at 1 day of incubation under low-nutrients and hypoxic conditions (Fig. 7c).

## Discussion

In between the period of transplantation and revascularization, islets are exposed to suboptimal conditions with low availability of oxygen and nutrients. Here we show, to our best knowledge for the first time, that under these circumstances not only apoptosis and necrosis but also autophagy and necroptosis occur in islets. Autophagy can be induced by the same factors that induce apoptosis, i.e. nutrient deprivation, but with different consequences. Apoptosis normally does not induce inflammation while autophagic bodies can interact with antigen presenting cells (APCs) to induce immune responses^19^. However, our data suggest that primary apoptosis in islet-cells may also lead to immune activation since we show that hypoxia-induced primary apoptosis in islets present the non-cleaved caspase-9 and −3 but not their cleaved products. This indicates an active process of cellular dead towards necrosis rather than secondary apoptosis^29^,^30^. This will lead to inflammation-inducing DAMPs. Thus, our data suggest that all cell-death processes including apoptosis in the islets may contribute to inflammation. The final result is always loss of functional cell-mass and release of inflammatory components from the intracellular space, *i.e.* DAMPs. This is to our opinion an important observation as strategies to prevent islet-loss by suppressing apoptosis or autophagy will have less effect than suppressing necrosis and necroptosis.

To determine the loss of islet-cells as the consequence of the harsh culture conditions, we first determined the DNA content of the islets at day 1 and 7. This was done initially by measuring the total-DNA and not by measuring nuclear DNA. In these initial experiments with total DNA we could not detect any DNA-reduction in the cultures even under very harsh low-oxygen and low-nutrition conditions. We contemplated that total DNA might be an inadequate measure for cell-numbers in islets due to presence of free DNA released by necrotic cells. Therefore we applied in the present study a two-step approach. We first isolated the nucleus from surviving cells and performed a DNA-quantification on the nuclei, representing intact cells. A comparison with total-DNA confirmed that total DNA did not reflect the number of surviving cells (data not shown) after which we only applied nuclear DNA for quantifying surviving islet cells. Notably, many studies might suffer from an overestimation of viable cells in islets as total-DNA is the golden standard in studies on glucose-induced insulin release^1^,^31^. Unfortunately we cannot do these types of measurements on small numbers of islets as the consequence of which we cannot express the DAMP production per unit of DNA.

Our study demonstrates that human pancreatic islets are potent producers of DAMPs such as dsDNA, uric acid, and HMGB1. HSP70, a potent inflammatory DAMP^17^, was not released by islets. The dsDNA is recognized by TLR9^18^,^32^,^33^ an inducer of antibodies and CD8+ T-cell responses^18^. Uric acid can be recognized by TLR2 and 4 but has also been reported to be responsible for destruction of beta-cells and suppression of basal insulin release^34^. HMBG1, a DNA transcriptional regulator, triggers immune responses by forming complexes with other molecules and binding combinations of PRRs such as TLR2/TLR4 and the receptor for advanced glycosylation end products (RAGE)^16^. The release of these DAMPs by islets was more pronounced in low-nutrient conditions than under hypoxia.

As many DAMPs are large molecules that cannot readily pass immunoisolating membranes we investigated the effect of encapsulation on DAMP-release and immune-activation. This reducing effect was profound but dependent on the type of DAMP studied. After encapsulation a reduction in DAMP-release in the medium was observed and a tenfold reduction in NF-κB activation. Concomitantly a reduction of HMGB1 release was observed. HMGB1 was undetectable after encapsulation of the islets. DsDNA was less reduced but retained as it was found throughout the capsules. Also nec-1 addition was an effective way to reduce DAMP release and immune activation in THP1 cells. The effects on cell loss were less pronounced than on DAMP release. This should be explained that some of the cells will probably die due to other cell-death processes than necroptosis^35^. Nec-1 suppresses the expression of the serine/threonine-protein kinases 1 and 3 (RIPK1 ad 3)^22^,^36^, *i.e.* two essential proteins for necroptosis induction. Our data demonstrate that this is an effective strategy as illustrated by a higher viability of the islets, a twofold reduction of NF-κB dependent immune activation under combined hypoxic and low-nutrient conditions.

## Conclusions

To our best knowledge, this is the first study dedicated to release of DAMPs by human pancreatic islets. Culturing human islets under the circumstances that islets may meet in the period between transplantation and vascularization induced the release of HMGB1, dsDNA, and uric acid. From these molecules it is known that they can induce a MyD88-TLR dependent immune response. Our data suggest that the use of immunoprotective membranes is efficacious in reducing DAMPs release and associated MyD88-dependent immune responses. Also, nec-1 administration might be an efficacious strategy to reduce DAMP release and associated immune activation in the immediate period after transplantation.

## Research Design and Methods

### Human islet isolation

Human islets were isolated from cadaveric pancreata at the Leiden University Medical Center, as previously described^37^,^38^. Islets were used if quality and/or number was insufficient for clinical application, according to national laws, and if research consent was available. All donor organs are coded (anonymized). Isolated islets were cultured as previously described^39^. All the methods and experimental protocols were approved and carried out in accordance with the Code of Proper Secondary Use of Human Tissue in The Netherlands as formulated by the Dutch Federation of Medical Scientific Societies. The Leiden University Medical Center has permission from the Dutch government to act as an organ bank for human islets. The organs, for which consent for research was obtained, were allocated by the Eurotransplant Foundation (Leiden, The Netherlands).

### Islet encapsulation

Intermediate-G alginate (ISP Alginates Ltd UK) has been purified and used^40^. Only highly purified alginates were applied as described before^41^. All the solutions were sterilized by filtration (0.2 μm pore size). Islets were suspended in purified alginate solution (3.4% w/v) at a concentration of 1000 islets/ml. The alginate-PLL-alginate (APA) capsules were formed as previously described^26^. The capsules had a final diameter of 650–700 μm.

In some experiments, for inhibition of necroptosis 100 μM of necrostatin-1 (nec-1) (Merck Chemicals, Darmstadt, Germany) was added to the alginate solution before encapsulation and incubation.

To measure DNA content, the capsules around the islets needed to be removed. To this end, the capsules were incubated with trypsin-EDTA-PBS buffer (TEP, 0.5% trypsin (v/v), 0.5 mM EDTA in PBS 1x pH 7.4) at 37 °C under constant shaking for 30 min.

### Experimental culture conditions

Islets (encapsulated or non-encapsulated) were exposed to different experimental conditions to mimic the conditions they may encounter *in vivo*. To mimic the relative low oxygen tensions after transplantation^9^,^42^, islets were cultured under hypoxic conditions (1% O_2_, 5% CO_2_, and 94% N_2_). Normoxic (20% O_2_, 5% CO_2_, and 75% N_2_) conditions served as control. Insufficient revascularization after implantation of islets can also lead to low nutrition^43^. To simulate low nutrition, islets were cultured without fetal calf serum (FCS) in CMRL 1066 medium (Life Technologies, NY, USA) supplemented with 8.3 mM glucose, 10 mM HEPES, 2 mM L-glutamine and 1% Penicilin/Streptomycin. As control served CMRL 1066 medium containing 10% FCS, 8.3 mM glucose, 10 mM HEPES, 2 mM L-glutamine and 1% Penicilin/Streptomycin.

Islets were cultured in a 24-well non-treated plate (Costar^®^, New York, USA). Each well contained 25 free or encapsulated islets in 0.8 ml of CMRL 1066 medium and were incubated in control or low-nutrient condition combined with normoxia or hypoxia.

### Measurement of specific danger/damage-associated molecular patterns (DAMPs)

To quantify the danger/damage-associated molecular patterns (DAMPs), ELISAs were used on supernatant of incubated islets. ELISAs for detecting High-Mobility Group Box 1 (HMGB1, Qayee-Bio, Shanghai, China), Heat Shock Protein 70 (HSP70, Donglin Sci & Tech, Wuxi/Jiangsu, China), double stranded DNA (dsDNA, BlueGene Biotech, Shanghai, China), and uric acid (Abcam®, Cambridge, UK) were performed according to standard protocols provided by the manufacturer.

### Screening for immunostimulation and identification of pattern recognition receptor activation

To determine the immunostimulatory capacity elicited by free or encapsulated islets, samples of the supernatant of islet-cultures were taken on day 1 and 7. Also, islets were collected for confocal microscopy analysis. The samples for immunostimulatory capacity were co-incubated with different cell lines (InvivoGen, Toulouse, France) that express pattern recognition receptors (PRRs) under the control of a SEAP reporter gene inducible by the transcription factors NF-κB/AP-1^25^,^26^,^44^ and contained additional inserts for the co-signaling molecules CD14 and MD2 to facilitate TLR-mediated responses^45^. In addition, we applied a THP1 cell expressing a non-functional form of the TLR adapter MyD88 (THP1-XBlue™-defMyD).

THP1-XBlue™-MD2-CD14 and -defMyD88 cells were suspended in fresh RPMI 1640 culture medium containing 2 mM L-glutamine, 1.5 g/l sodium bicarbonate, 4.5 g/l, 10 mM HEPES and 1.0 mM sodium pyruvate, and supplemented with 10% fetal bovine serum (deactivated phosphatases), 100 μg/ml Normocin™, Pen-Strep (50 U/ml–50 μg/ml) at 1 × 10^6^ cells/ml and plated in 96-well plates. Each well was stimulated with samples of supernatant from naked or encapsulated islets in control or starvation conditions and 20% or 1% of O_2_ and cultured overnight at 37 °C and 5% CO_2_. As positive controls, lipopolysaccharide from *Escherichia coli* K12 strain (LPS-EK Ultrapure 10 μg/ml, InvivoGen, Toulouse, France) was used for the THP1-XBlue™-MD2-CD14 cell line and L-Ala-γ-D-Glu-mDAP (Tri-DAP 10 μg/ml, InvivoGen,Toulouse, France) for theTHP1-XBlue™-defMyD88 cell line. In both cell lines RPMI 1640 culture medium was used as a negative control. Production of SEAP was quantified by using QUANTI-Blue™ (InvivoGen, Toulouse, France) a medium for detection and quantification of alkaline phosphatase. QUANTI-Blue™ medium change to a purple- blue color in the presence of SEAP. An aliquot of QUANTI-Blue™ (200 μl) was brought in a new flat bottom 96-well plate with 20 μl of supernatant from the stimulated cell-lines for 45 min at 37 °C and SEAP activity, representing activation of NF-κB/AP-1, was measured at a wavelength of 650 nm on a VersaMax microplate reader (Molecular Devices GmbH, Biberach an der Riss, Germany) using SoftMax Pro Data Acquisition & Analysis Software^15^,^25^,^40^.

### Confocal analysis

Apoptosis was measured using Annexin V, Alexa Fluor® (InvitroGen Life Technologies^TM^, NY, USA). Collected naked or encapsulated islets were incubated for 30 min with Annexin V (3 μl per 1 ml of cell culture) at room temperature avoiding light. After incubation, the islets were washed five times with KRH. Fluorescent confocal microscopy was measured at an emission wavelength of 665 nm using a Leica TCS SP2 AOBS confocal microscope (Wetzlar, Germany) equipped with an objective HC PL APO CS 10x/0,30, dry immersion, and working distance of 11 mm. Data were analyzed using Imaris® x64 version 7.6.4 software and ImageJ 1.47.

### Measurement of autophagy in islets

Autophagic cells were identified using a Premo^TM^ Autophagy expression kit, time-resolved fluorescence resonance energy transfer of terbium-labeled LC3B antibodies (Tb/GFP TR-FRET LC3B) from Molecular Probes® (Life Technologies^TM^, NY, USA)^27^. As positive control we applied the lysosomal inhibitor chloroquine (CQ) (half-dilutions from 100-0 μM) to block the turnover of autophagosomes. Islets cultured under control conditions (complete CMRL 1066, 10% FCS v/v, 20% O_2_) served as negative control.

The islets were tested after 30 min exposure to low-nutrition conditions and/or hypoxic conditions (CMRL 1066 without FCS, 1% O_2_) following the manufacturer protocol. The assay plate was read using a Thermo Scientific Varioskan® reader for fluorescence (MA, USA) with an excitation wavelength of 332/12 nm, emission 1 of 488/12 nm, and emission 2 of 518/12 using 200 flashes for the acceptor channel (518 nm) and 100 flashes for the terbium channel (488 nm). For each well, the TR-FRET emission ratio (518 nm/488 nm) was calculated by dividing the acceptor emission value by the donor emission value. To normalize data, assay window values were determined by dividing the TR-FRET emission ration by the CQ-treated emission ratio values by the average emission ratio of the baseline control^27^.

### Western blot

Human islets were lysed using a radio-immunoprecipitation assay (RIPA) buffer 10x (Millipore, The Netherlands) supplemented with protease inhibitors. The protein concentration was determined using the BCA (bicinchoninic acid) Protein Assay (Thermo scientific, MA USA). An equal amount of protein was loaded on a 10% polyacrylamide (PAA) gel, separated and transferred to a nitrocellulose Immobilon®-FM transfer membrane (Millipore, The Netherlands). The membrane was blocked with Odyssey® blocking buffer (Li-cor, The Netherlands) for 1 hr at room temperature and then incubated with the primary antibody in 1x PBS with 0.1% Tween overnight at 4 °C. A caspase-3 human specific antibody (Cell Signaling Technology, Leiden, The Netherlands) and a caspase-9 human specific antibody (Cell Signaling Technology, Leiden, The Netherlands) were used to detect endogenous levels of full-length caspase-3 or −9 (32 and 47 kDa respectively) and large fragments of caspase-3 or −9. The membrane was washed with a phosphate-buffer saline with Tween (PBS-T) and incubated for 1 hr with the secondary antibody IRDye 800CW (Odyssey, Li-cor, The Netherlands) in the dark. Finally, β-actin rabbit antibody (Cell Signaling Technology, Leiden, The Netherlands) was used as housekeeping protein. Primary antibodies were used at a dilution of 1:1000, and secondary antibody at a dilution of 1:2000.

### Nuclei isolation and DNA content measurement

A Nuclei EZ prep nuclei isolation kit (Sigma-Aldrich, MO, USA) was used to isolate nuclei and count only nuclear DNA in order to quantify the amount of live cells after the different culture conditions. Islets were centrifuged at 500 G for 5 min. The supernatant was removed and the cells were washed with PBS the supernatant was discarded and 1 ml of cold Nuclei EZ prep nuclei isolation lysis buffer was added. Cells were vortexed and incubated at 4 °C for 5 min. The remaining cellular material was centrifuged at 500 G for 5 min to sediment nuclei and supernatant with other cellular components was discarded. Isolated nuclei were subsequently used to quantify the DNA content.

Quantification of DNA in islet nuclei was performed using a Quant-iT™ PicoGreen® dsDNA Assay Kit (Live Technologies, The Netherlands)^46^. The nuclei were incubated with 100 μl of TE buffer (200 mM Tris-HCl, 20 mM EDTA, pH 7.5). After adding the solution nuclei were sonicated for 10 sec at 30% power and plated in a 96 well-plate. Reagent for fluorescent detection was prepared by making a 200-fold dilution of the Quanti-iT PicoGreen reagent in TE buffer and addition of 100 μl to the samples. Quantification of dsDNA was done in a fluorescence microplate reader Thermo Scientific Varioskan® (Thermo Scientific, MO, USA) at excitation and emission wavelengths of 480 and 520 nm respectively.

### Statistical analysis

Values are expressed as median ± IQR. Statistical comparisons were performed using the Mann–Whitney U test to compare the outcomes of the nonparametric, unmatched treatments of controls and islets cultured under low nutrient conditions. Values of *p *< 0.05 were considered to be statistically significant.

## Additional Information

**How to cite this article**: Paredes-Juarez, G. A. *et al.* DAMP production by human islets under low oxygen and nutrients in the presence or absence of an immunoisolating-capsule and necrostatin-1. *Sci. Rep.* **5**, 14623; doi: 10.1038/srep14623 (2015).

## Supplementary Material

Supplementary Information
