# Supplementary material for: DAMP production by human islets under low oxygen and nutrients in the presence or absence of an immunoisolating-capsule and necrostatin-1
Source: Sci Rep. 2015 Sep 30;5:14623. doi: 10.1038/srep14623 (PMC4588515; doi:10.1038/srep14623)
Supplement: Supplementary Information [file srep14623-s1.pdf]

DAMP production by human islets under low oxygen and nutrients in the presence or absence of an immunoisolating-capsule and necrostatin-1

Genaro A. Paredes-Juarez[1,*]; Neha M. Sahasrabudhe[1]; Reina S. Tjoelker[1]; Bart J. de Haan[1]; Marten A. Engelse[2]; Eelco J.P. de Koning[2]; Marijke M. Faas[1]; and Paul de Vos[1]

Authors' information:

[1] University of Groningen, University Medical Center Groningen, Department of Pathology and Medical Biology, Section of Immunoendocrinology, Groningen, 9713GZ, The Netherlands.

[2] Department of Nephrology, Leiden University Medical Center, Leiden, 2300 RC, The Netherlands.

* Correspondence:

G. A. Paredes-Juarez, M.Sc.

University of Groningen

University Medical Center Groningen

Department of Pathology and Medical Biology

Section of Immunoendocrinology

Hanzeplein 1, EA11, 9700 RB

Groningen The Netherlands

tel direct: 31-50-3611045

tel secr: 31-50-3618043

fax: 31-50-3619911

G.Paredes@umcg.nl

Running title: DAMP production by encapsulated islets with nec1

SUPPLEMENTARY DATA

(Legend to Figure S1)

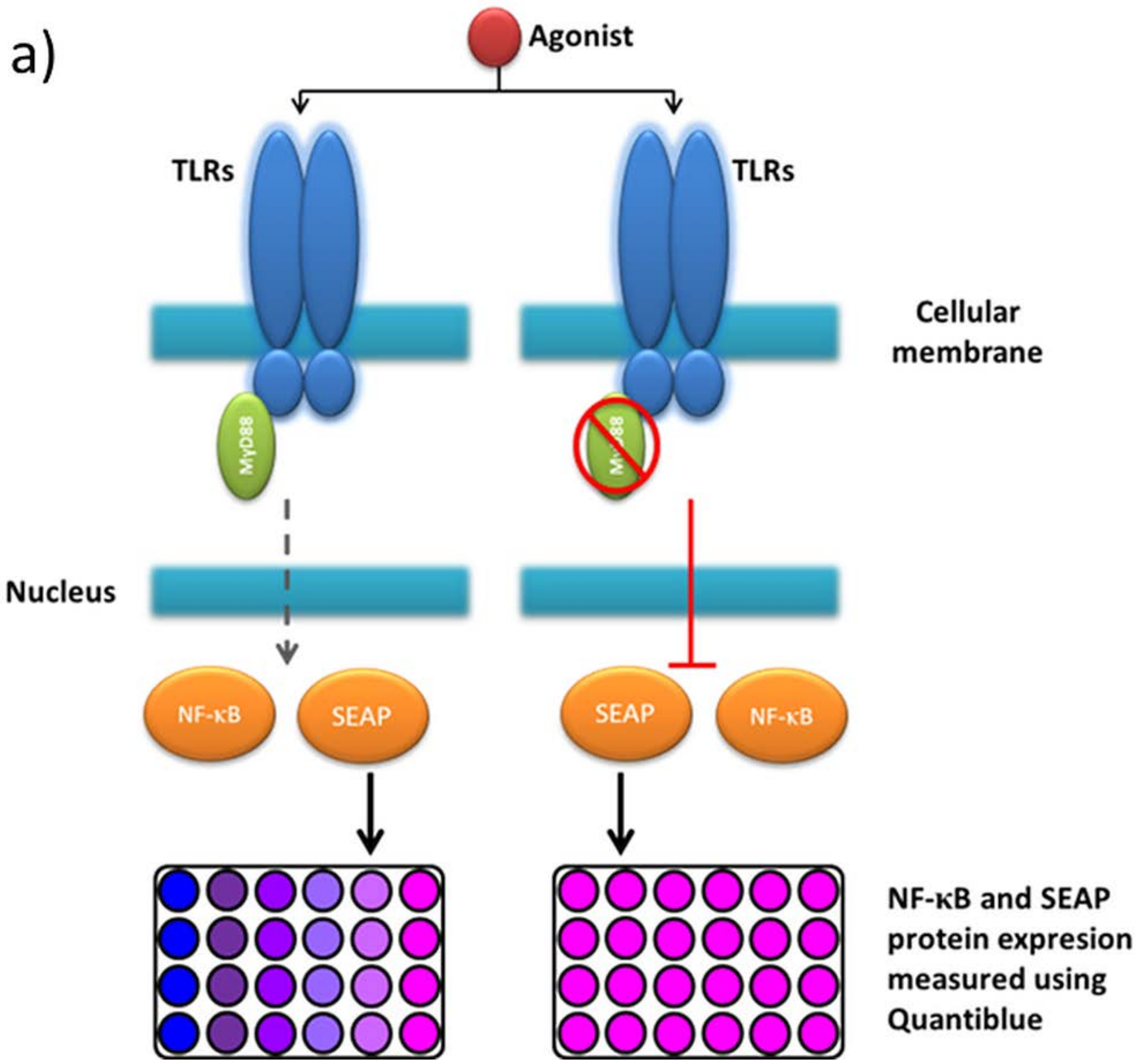

Figure S1. Assay to determine MyD88 and TLR dependency of activation of THP1 cells.

Agonists of pattern recognition receptors (PRRs) activate the transcription NF- κ B that is coupled to a secreted embryonic alkaline phosphatase (SEAP). SEAP is quantifiable with a colorimetric detection medium (left). To determine whether the activation is TLR dependent, a THP1 cell line with a truncated non-functional myeloid differentiation primary response gene (88) (MyD88) adaptor molecule was applied (right). An activation of NF- κ B in THP1 but not in THP1 with a deficient MyD88 indicates a TLR dependency.

(Legend to Figure S2)

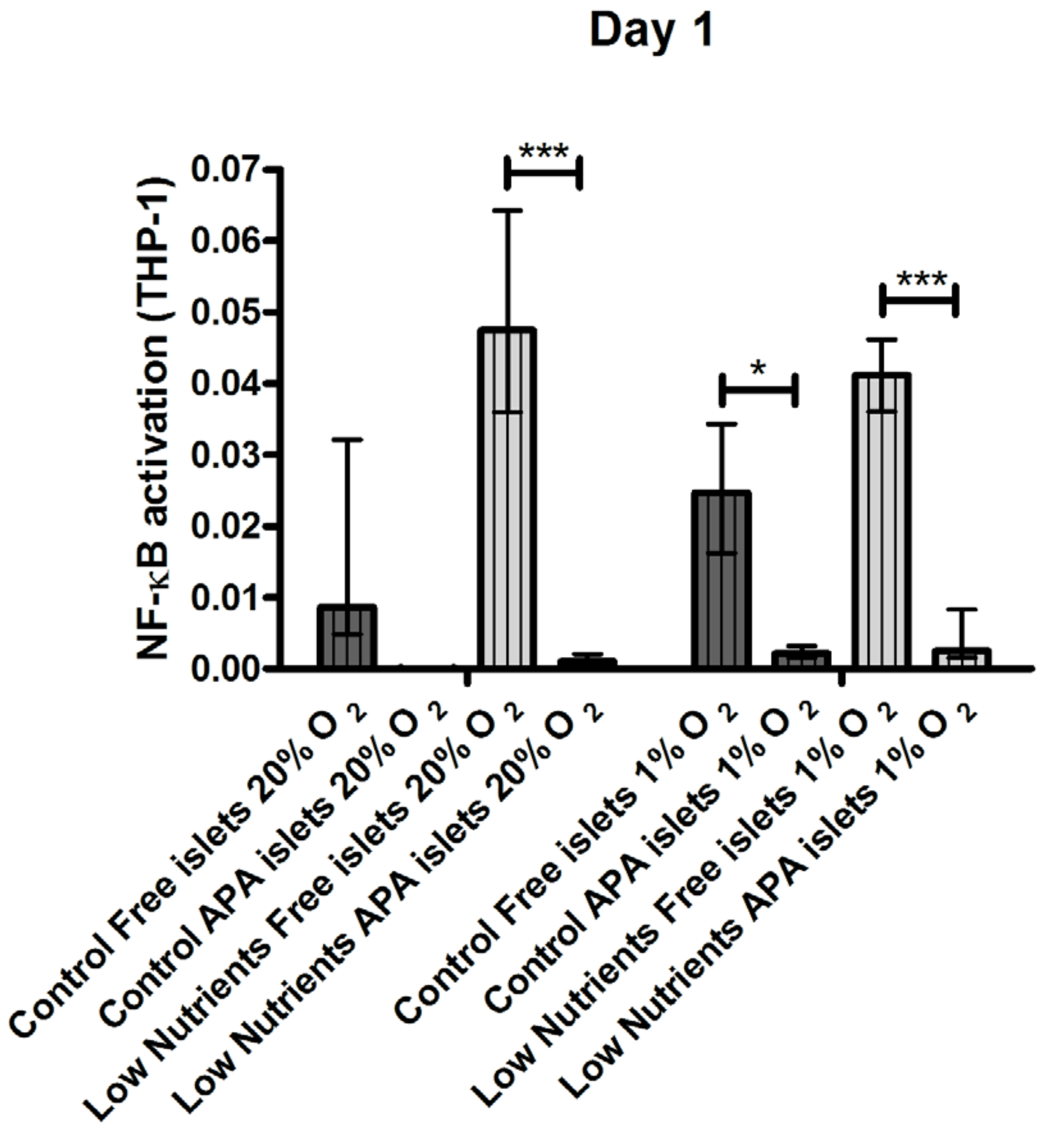

Figure S2. Reduction of NF-κB activation after encapsulation of human islets. The THP1 cell line with a functional MyD88 molecule was stimulated with supernatant of free isolated human islets or encapsulated islets in tradition APA system after 1 day of incubation under control or low nutrients at 20% or 1% oxygen conditions. Values are presented as median ± IQR (n = 4 separate batches of human islets) a $p < 0.05$ was consider statistical significant ($p < 0.05$, *; $p < 0.001$, ***). LPS (10 μg/ml) was used as positive control for THP1 cell line.

(Legend to Figure S3)

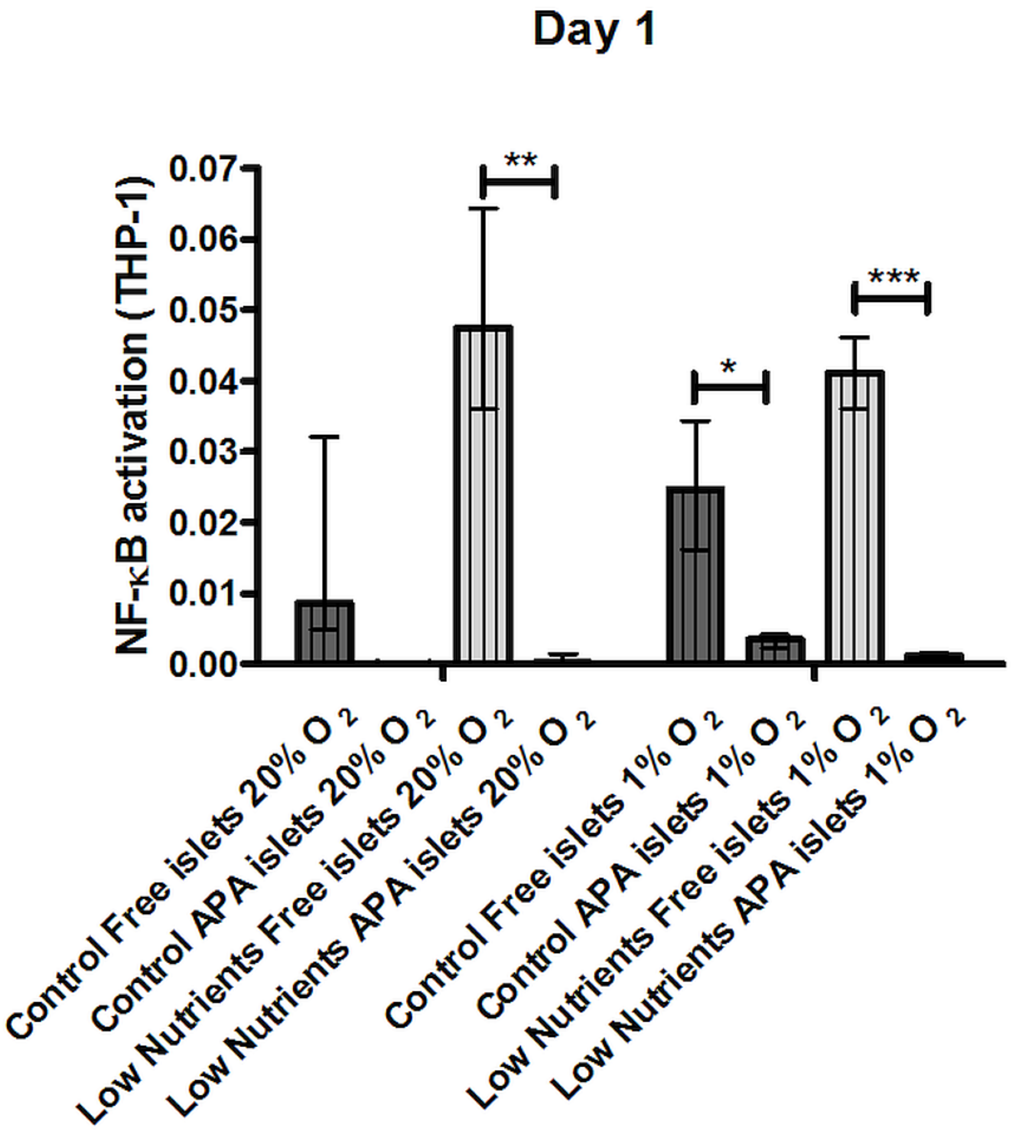

Figure S3. Reduction of NF- κ B activation by addition of nec-1 in encapsulated human islets.

The THP1 cell line with a functional MyD88 molecule was stimulated with supernatant of encapsulated islets in APA capsules or with nec-1 incubated for 1 day under control or low nutrients at 20% or 1% oxygen conditions. Values are presented as median ± IQR (n = 4 separate batches of human islets) a $p < 0.05$ was consider significant ($p < 0.05$, *; $p < 0.01$, **; $p < 0.001$, ***). LPS (10 µg/ml) was used as positive control for THP1 cell line.
